# Melatonin protects against focal cerebral ischemia-reperfusion injury in diabetic mice by ameliorating mitochondrial impairments: involvement of the Akt-SIRT3-SOD2 signaling pathway

**DOI:** 10.18632/aging.203137

**Published:** 2021-06-11

**Authors:** Lian Liu, Quan Cao, Wenwei Gao, Bingyu Li, Zhongyuan Xia, Bo Zhao

**Affiliations:** 1 Department of Anesthesiology, Renmin Hospital of Wuhan University, Wuhan, Hubei 430060, China; 2 Department of Ultrasound Imaging, Renmin Hospital of Wuhan University, Wuhan, Hubei 430060, China; 3 Critical Care Medicine, Renmin Hospital of Wuhan University, Wuhan, Hubei 430060, China

**Keywords:** melatonin, diabetes, ischemic stroke, mitochondria, therapy

## Abstract

Diabetic patients are more vulnerable to cerebral ischemia-reperfusion (CIR) injury and have a worse prognosis and higher mortality after ischemic stroke than non-diabetic counterparts. Melatonin can exert neuroprotective effects against CIR injury in nondiabetic animal models. However, its effects on diabetic CIR injury and the underlying mechanisms remain unclarified. Herein, we found that melatonin administration improved neurological deficit, cerebral infarct volume, brain edema, and cell viability, reduced mitochondrial swelling, reactive oxygen species generation, and cytoplasmic cytochrome C release, and increased mitochondrial antioxidant enzymes activities, adenosine triphosphate production, and mitochondrial membrane potential in both streptozotocin-induced diabetic mice and high glucose-treated HT22 cells. Importantly, melatonin also activated protein kinase B (Akt) and sirtuin 3 (SIRT3)/superoxide dismutase 2 (SOD2) signaling and upregulated mitochondrial biogenesis-related transcription factors. However, these effects were largely attenuated by LY294002 (a specific Akt signaling blocker) administration. Additionally, 3-TYP (a selective SIRT3 inhibitor) and SIRT3 siRNA inhibited the above protective effects of melatonin as well as the upregulation of SIRT3 and the decrease of SOD2 acetylation but did not affect the p-Akt/Akt ratio. Overall, we demonstrate that melatonin can alleviate CIR injury in diabetic mice by activating Akt-SIRT3-SOD2 signaling and subsequently improving mitochondrial damage.

## INTRODUCTION

Stroke is the chief cause of physical and intellectual disability in adults and remains the leading cause of death in developed countries [1]. Diabetes increases the vulnerability and fragility of brain vessels, which increases the risk of ischemic stroke by more than 3.35-fold [2, 3]. Diabetic patients are more vulnerable to cerebral ischemia-reperfusion (CIR) injury [4, 5]. Epidemiological studies have shown that compared with non-diabetic patients, diabetic patients have a worse vascular prognosis, higher in-hospital mortality, and slower functional recovery after stroke [6, 7]. Unfortunately, the classic treatment for non-diabetic stroke patients with thrombolysis leads to an increased incidence of cerebral hemorrhage and a worsening neurological outcome when applied to patients with diabetes [8]. Several cell-based therapies, such as bone marrow stromal cells, can improve functional recovery after stroke in non-diabetic individuals. However, cell-based therapies increase brain hemorrhage transformation and induce cerebral arteriosclerosis-like changes in individuals with diabetic stroke [9, 10]. Therefore, it is of great clinical significance to elucidate the pathogenesis of diabetes complicated with ischemic stroke and explore effective prevention and treatment strategies.

The initial event of CIR injury is reactive oxygen species (ROS) burst production, which causes the oxidation of cellular proteins, DNA, and lipids [11]. Mitochondria are abundant in the brain, which is the chief source of cerebral intracellular ROS, and are particularly vulnerable to hypoxia and ischemia [12]. Mitochondrial insults lead to dysregulation of ROS homeostasis, which further leads to mitochondrial damage and continues a vicious cycle [13, 14]. Evidence shows that ischemic neuronal injury is particularly intensified during reperfusion due to impairment of mitochondria [15, 16]. Numerous pro-survival cascades, such as antioxidant enzyme activities and mitochondrial biogenesis are inhibited during reperfusion. The inhibition of the pro-survival cascades increases neuronal cell death and aggravates CIR injury [17, 18]. Additionally, chronic hyperglycemia in diabetes aggravates hemorrhagic transformation after ischemic stroke by mitochondrial defects-induced endothelial cell apoptosis [19]. Therefore, mitochondria-targeting therapy may be a potential therapeutic strategy for diabetic patients complicated with ischemic stroke.

Melatonin is a naturally synthesized hormone with a high local concentration in the brain and cerebrospinal fluid [20]. The synthesis and secretion of melatonin significantly decrease with aging, the relative deficiency of melatonin may be related to the pathophysiology of age-related neurological diseases [21, 22]. Importantly, increasing studies have confirmed the neuroprotective effects of melatonin in nondiabetic animal models with CIR injury [23, 24]. Moreover, melatonin contributes to maintaining mitochondrial homeostasis and protecting against mitochondria damage under various pathological conditions, including diabetes [25, 26]. However, whether melatonin protects against mitochondrial damage in the diabetic brain following CIR injury and the underlying mechanisms remain unknown.

Notedly, protein kinase B (PKB, also known as Akt) is a well-established pro-survival signaling molecule resistant to oxidative damage and mitochondrial insults in the brain [27, 28]. Previously, we and other scholars have demonstrated that Akt signaling activation protected the brain against CIR injury in nondiabetic animal models [29–31]. Sirtuin 3 (SIRT3) is the primary mitochondrial sirtuin in the brain [32]. Recently, SIRT3 has been reported to maintain ROS homeostasis by deacetylating and activating the antioxidant enzyme superoxide dismutase 2 (SOD2), which can convert harmful superoxide free radicals into nontoxic oxygen or hydrogen peroxide [33]. Emerging evidence shows that SIRT3/SOD2 signaling activation prevents oxidative stress and mitochondrial damage in multiple pathological conditions [34, 35]. Interestingly, a recent study reported that melatonin attenuates hepatocytes damage by inhibiting mitochondrial stress and activating the Akt-Sirt3 signaling pathway [36]. Therefore, we hypothesized that melatonin might exert protective effects in diabetes complicated with CIR injury by alleviating mitochondrial defects through activating the Akt-SIRT3-SOD2 signaling pathway.

## RESULTS

### Effects of melatonin on the cerebral infarct volume, neurological deficits, and brain edema in diabetic mice following CIR

**Figure 1:**
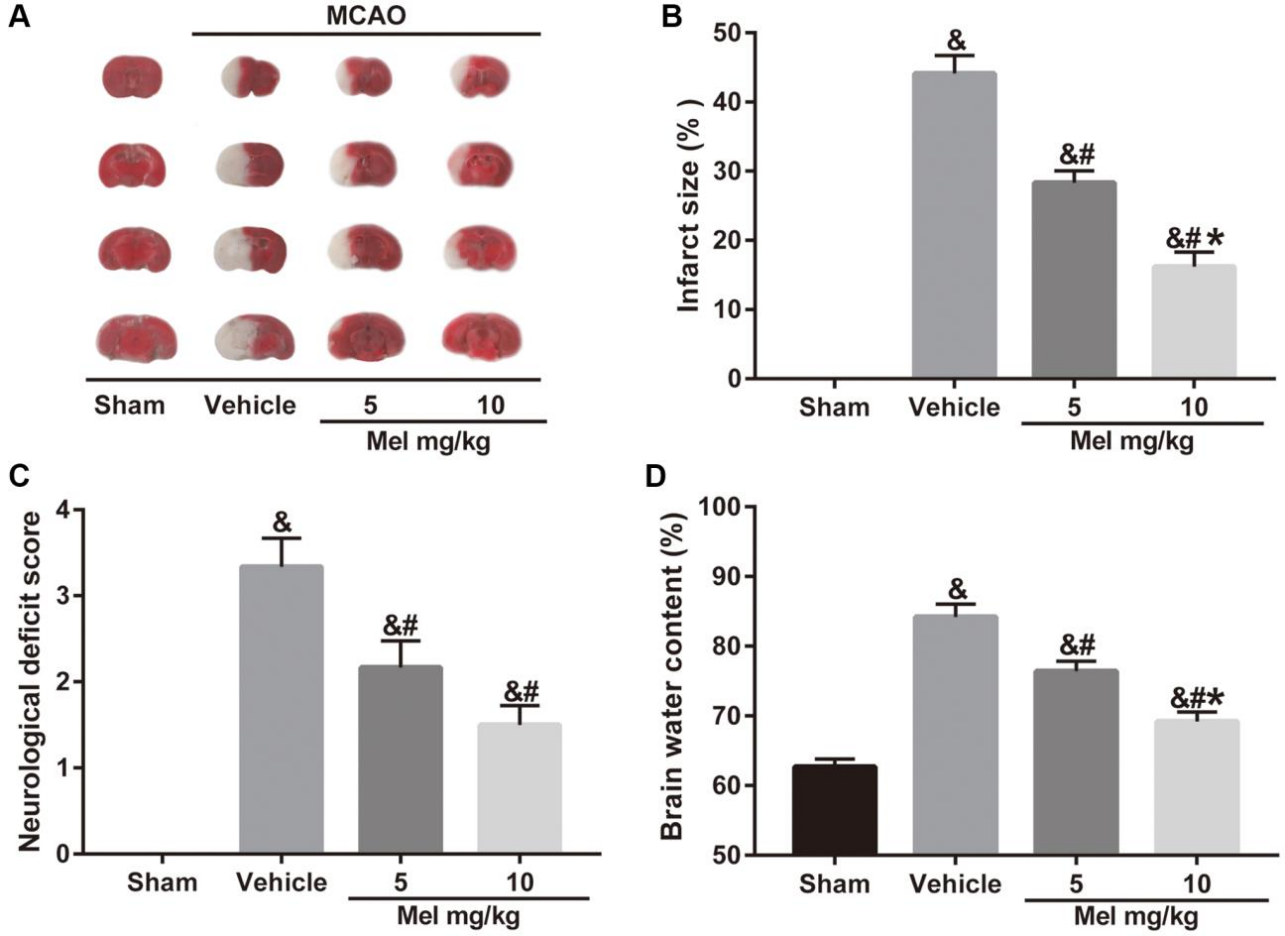
**Effects of melatonin on cerebral infarct volume, neurological function, and brain water content in diabetic mice with CIR injury.** (**A**) TTC staining of brain sections taken from diabetic mice with CIR injury. (**B**, **C**, **D**) Effects of melatonin at different concentrations on the infarct volume, neurological score, and brain water content in diabetic mice with CIR injury. Data were presented as the mean ± SEM (*n* = 6). ^&^*p* < 0.05 vs. sham group; ^#^*p* < 0.05 vs. vehicle group; ^*^p < 0.05 vs. melatonin (5 mg/kg).

To examine whether melatonin has a neuroprotective effect in diabetic mice following CIR injury, we assessed neurobehavioral outcomes, cerebral infarct volume, and brain edema. Neurological scoring was performed 24 h after middle cerebral artery occlusion (MCAO). Then, the mice were killed and the brains were quickly isolated, sliced, and stained with TTC. The results showed that administration of melatonin (5 and 10 mg/kg) significantly reduced the cerebral infarct volume and neurological deficits compared with the vehicle group (Figure 1A–1C). Similarly, compared with the vehicle group, melatonin (5 and 10 mg/kg) also alleviated brain edema (Figure 1D). Additionally, the 10 mg/kg dose had the optimal protective effect and was selected for subsequent experiments. The results enabled us to believe that melatonin exerts neuroprotective effects in diabetes complicated with CIR injury.

### Effects of SIRT3/SOD2 signaling on the melatonin-mediated alleviation of mitochondrial oxidative stress induced by CIR in diabetic mice

**Figure 2:**
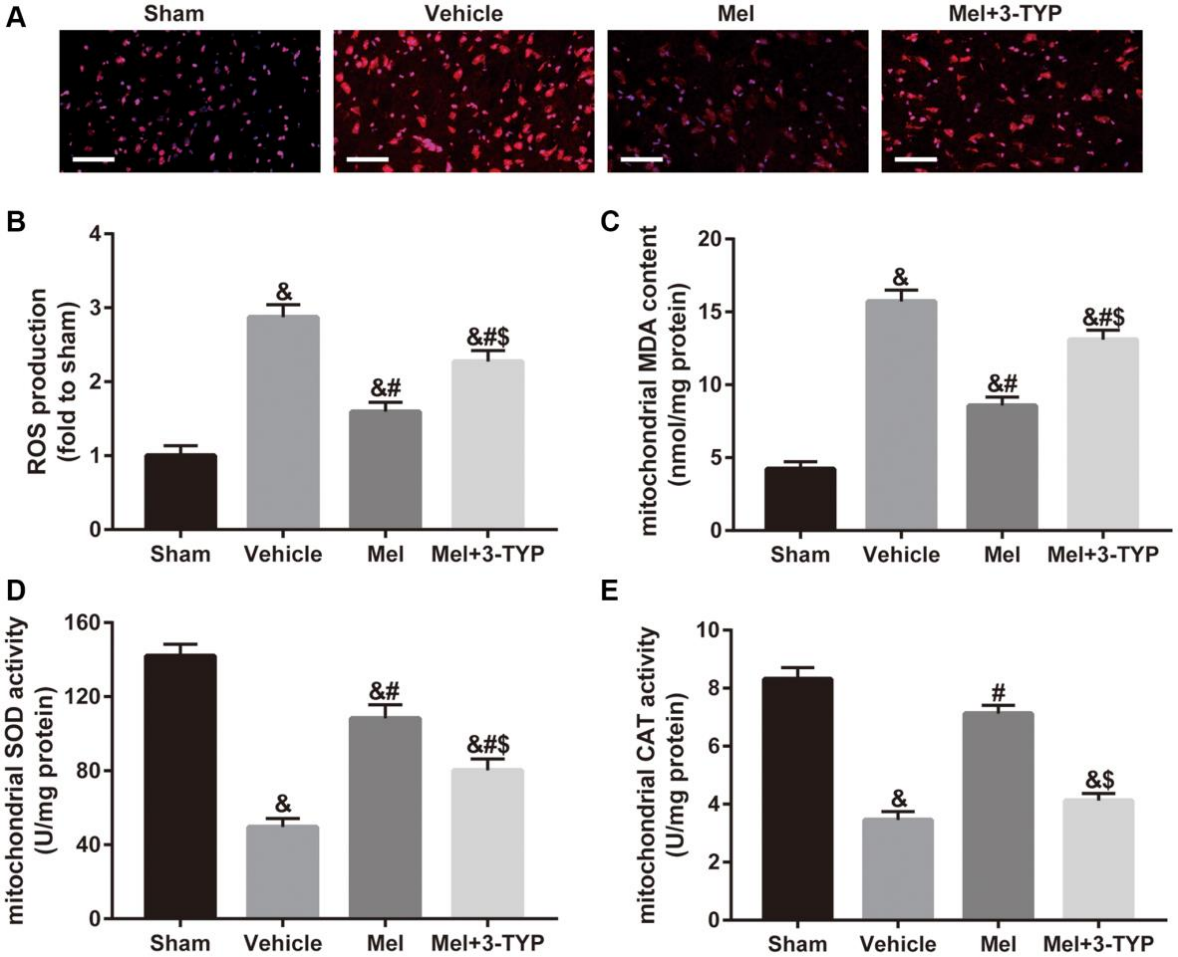
**Effects of melatonin on mitochondrial oxidative stress in diabetic mice with CIR injury.** (**A**) Changes in the production of reactive oxygen species (ROS) were revealed by DHE staining (×400). Scale bar = 50 μm. (**B**) Quantification of ROS production in the brain. (**C**–**E**) Quantitative analysis of levels of mitochondrial malondialdehyde (MDA), superoxide dismutase (SOD), and catalase (CAT) were quantified by using commercial kits. Data were presented as the mean ± SEM (*n* = 6). ^&^*p* < 0.05 vs. sham group; ^#^*p* < 0.05 vs. vehicle group; ^$^*p* < 0.05 vs. Mel group.

To determine the underlying mechanisms of melatonin on CIR in the diabetic state, we evaluated mitochondrial oxidative stress in our *in vivo* experiment. Since mitochondria are the main intracellular sources of ROS [12], we first measured ROS production in brain tissue The results showed that melatonin significantly reduced ROS level while the above effect was significantly weakened after the use of SIRT3 specific inhibitor 3-TYP (Figure 2A and 2B). Additionally, we evaluated mitochondrial malondialdehyde (MDA) content, superoxide dismutase (SOD) activity, and catalase (CAT) activity in these experimental groups. MDA is a frequently used membrane lipid peroxidation hallmark, while SOD and CAT are anti-oxidative enzymes that can remove free radicals produced during the metabolic process and reduce damage from oxygen free radicals [37]. As shown in Figure 2C–2E, melatonin effectively improved mitochondrial SOD and CAT activity, and reduced mitochondrial MDA generation. Consistently, 3-TYP also blunted these effects.

### Effects of SIRT3/SOD2 signaling on the melatonin-mediated amelioration of mitochondrial defects in diabetic mice following CIR

**Figure 3:**
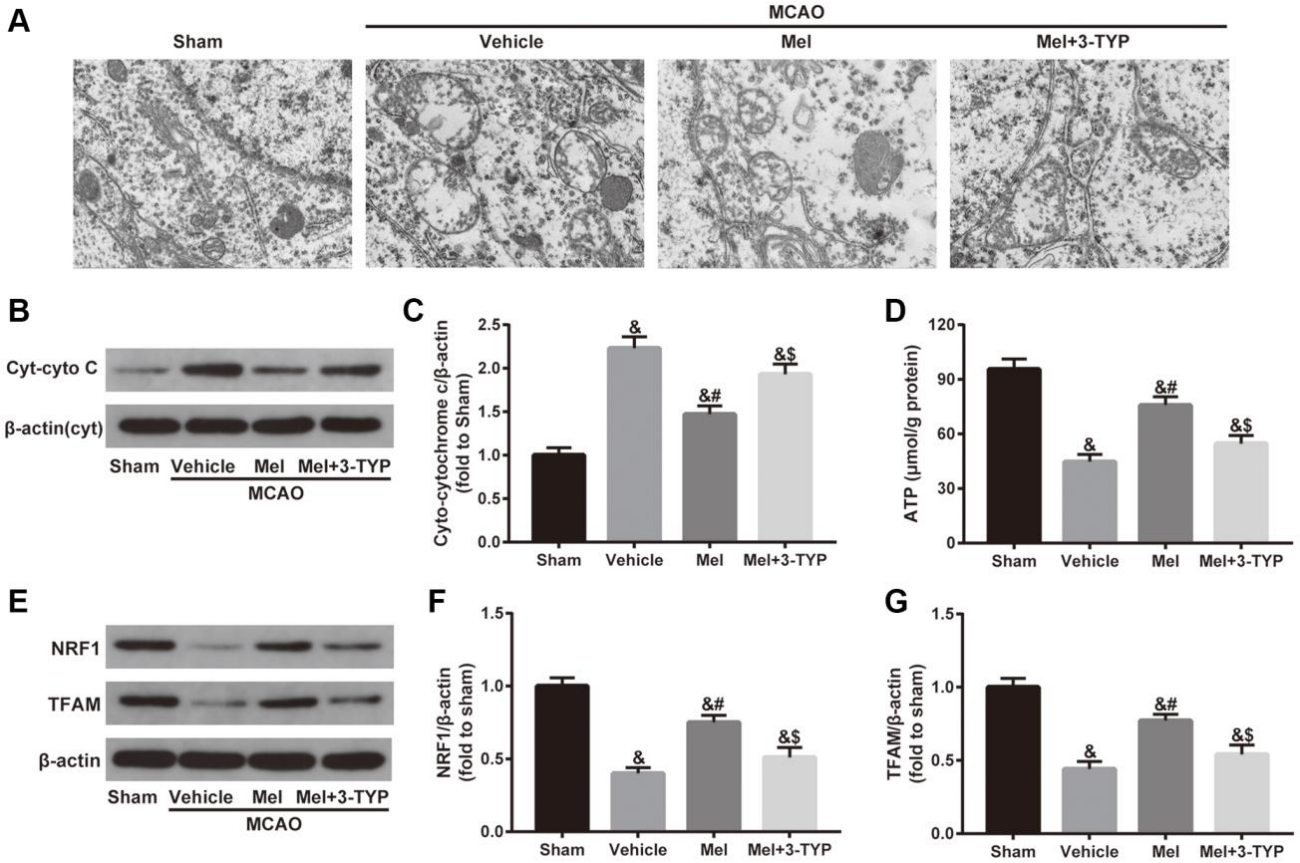
**Effects of melatonin on mitochondrial defects in diabetic mice with CIR injury.** (**A**) Representative images of the ultrastructural changes of mitochondria under electron microscopy (×1.2k). (**B**) Representative images for Cytoplasmic cytochrome C (Cyt-cyto C) expression detected by Western blot. (**C**) Quantitative analysis of the Cyt-cyto C protein levels. (**D**) Quantitative analysis of mitochondrial ATP content. (**E**) Representative images for NRF1 and TFAM expression detected by Western blot. (**F**, **G**) Quantitative analysis of the NRF1 and TFAM levels. Data were presented as the mean ± SEM (*n* = 6). ^&^*p* < 0.05 vs. sham group; ^#^*p* < 0.05 vs. vehicle group; ^$^*p* < 0.05 vs. Mel group.

As shown in Figure 3A, mitochondria in the sham group showed normal morphological structure with integrated mitochondrial crest while mitochondria in the vehicle group showed serious vacuolization and swelling. Melatonin significantly improved mitochondrial vacuolization and swelling while the beneficial effects of melatonin were attenuated by 3-TYP. Next, we evaluated the integrity of mitochondria by detecting the expression of cytoplasmic cytochrome C (Cyt-cyto C) (Figure 3B and 3C). Cytochrome C is an important component of the electron transport chain in mitochondria. Once the integrity of mitochondria is impaired, the expression level of Cyt-cyto C would be significantly increased [38]. Our results showed that melatonin treatment significantly reduced CIR-induced upregulation of Cyt-cyto C, suggesting that melatonin exerted beneficial effects in maintaining the integrity of mitochondria but the effects were significantly blunted by 3-TYP. Furthermore, melatonin significantly improved CIR-induced mitochondrial adenosine triphosphate (ATP) deficiency, which was also prominently attenuated by 3-TYP (Figure 3D). Besides, we measured the expression of nuclear respiratory factor 1(NRF1) and mitochondrial transcription factor A (TFAM), two important mitochondrial biogenesis factors. The results showed that melatonin significantly increased their expressions in the Mel group compared with those in the vehicle group. Consistently, 3-TYP largely weakened these effects (Figure 3E–3G).

### Effects of Akt signaling on SIRT3/SOD2 signaling in the neuroprotection of melatonin against CIR injure in diabetic mice

**Figure 4:**
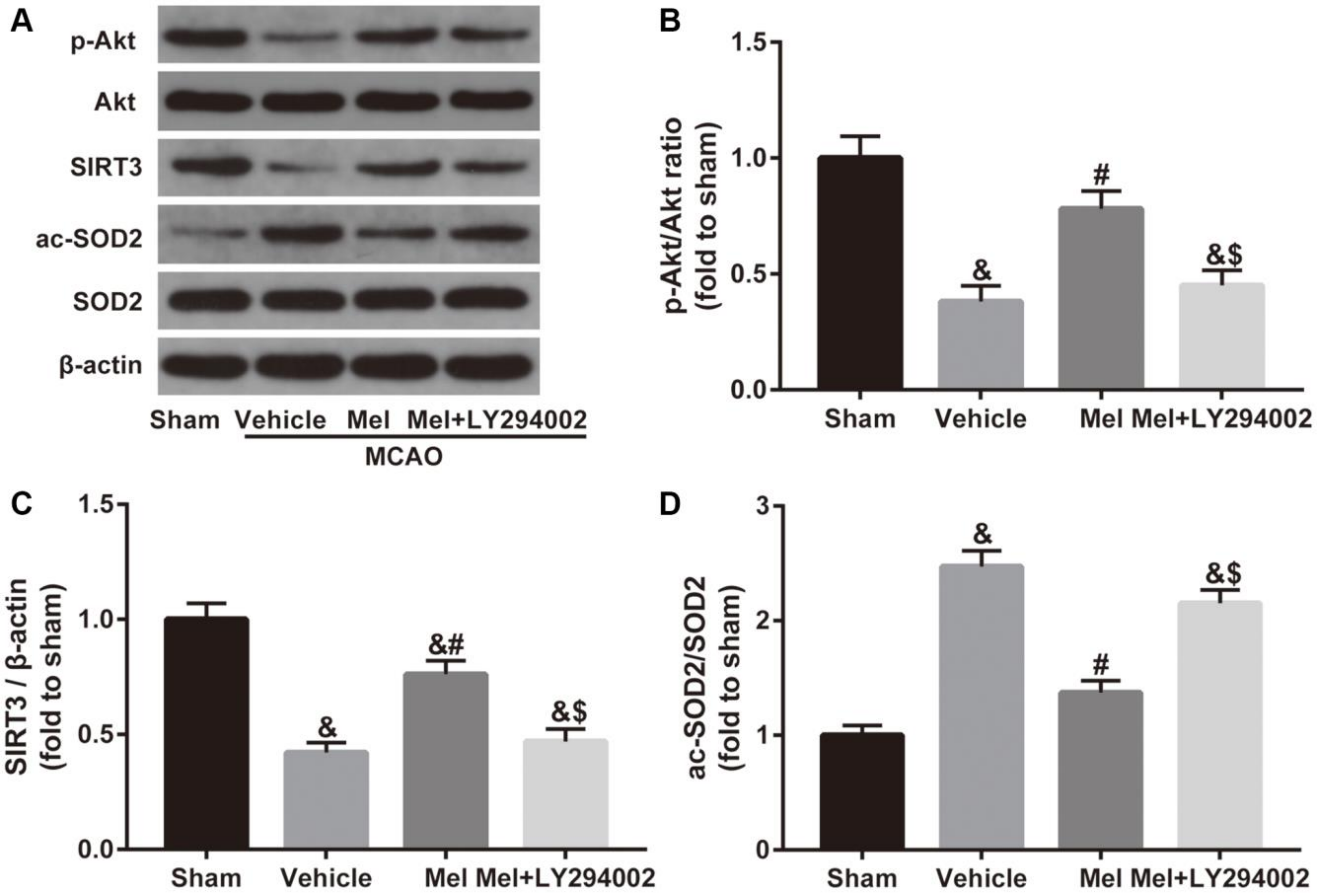
**Effects of Akt signaling on the SIRT3/SOD2 pathway in the neuroprotective effects of melatonin in diabetic mice with CIR injury.** (**A**) Representative images for Akt phosphorylation, SIRT3 expression, and SOD2 acetylation detected by Western blot. (**B**–**D**) Quantitative analysis of the ratio of p-Akt/Akt, expression of SIRT3 and the ratio of ac-SOD2/SOD2 in ischemic cortical tissue. Data were presented as the mean ± SEM (*n* = 6). ^&^*p* < 0.05 vs. sham group; ^#^*p* < 0.05 vs. vehicle group; ^$^*p* < 0.05 vs. Mel group.

To investigate the mechanism of melatonin-induced SIRT3/SOD2 signaling activation, the Akt signaling was studied further. As shown in Figure 4, CIR induced a reduced phosphorylation level of Akt (p-Akt/Akt ratio), accompanied by decreased SIRT3 expression and increased acetylation of SOD2 (ac-SOD2/SOD2 ratio). In contrast, melatonin treatment significantly increased the phosphorylation of Akt and the expression of SIRT3 and reduced the acetylation of SOD2. Notably, inactivation of the Akt pathway using LY294002 mediated a reduction in the phosphorylation of Akt and also caused a decrease in the SIRT3 expression and induced an elevation of acetylation of SOD2 in the presence of melatonin. Collectively, it suggested that Akt is required for the activation of SIRT3-SOD2 signaling by melatonin in the CIR-injured diabetic brain.

### Effects of Akt signaling and SIRT3/SOD2 signaling in simulated ischemia-reperfusion (SIR)-induced cell injury in high glucose treated-HT22 cells

**Figure 5:**
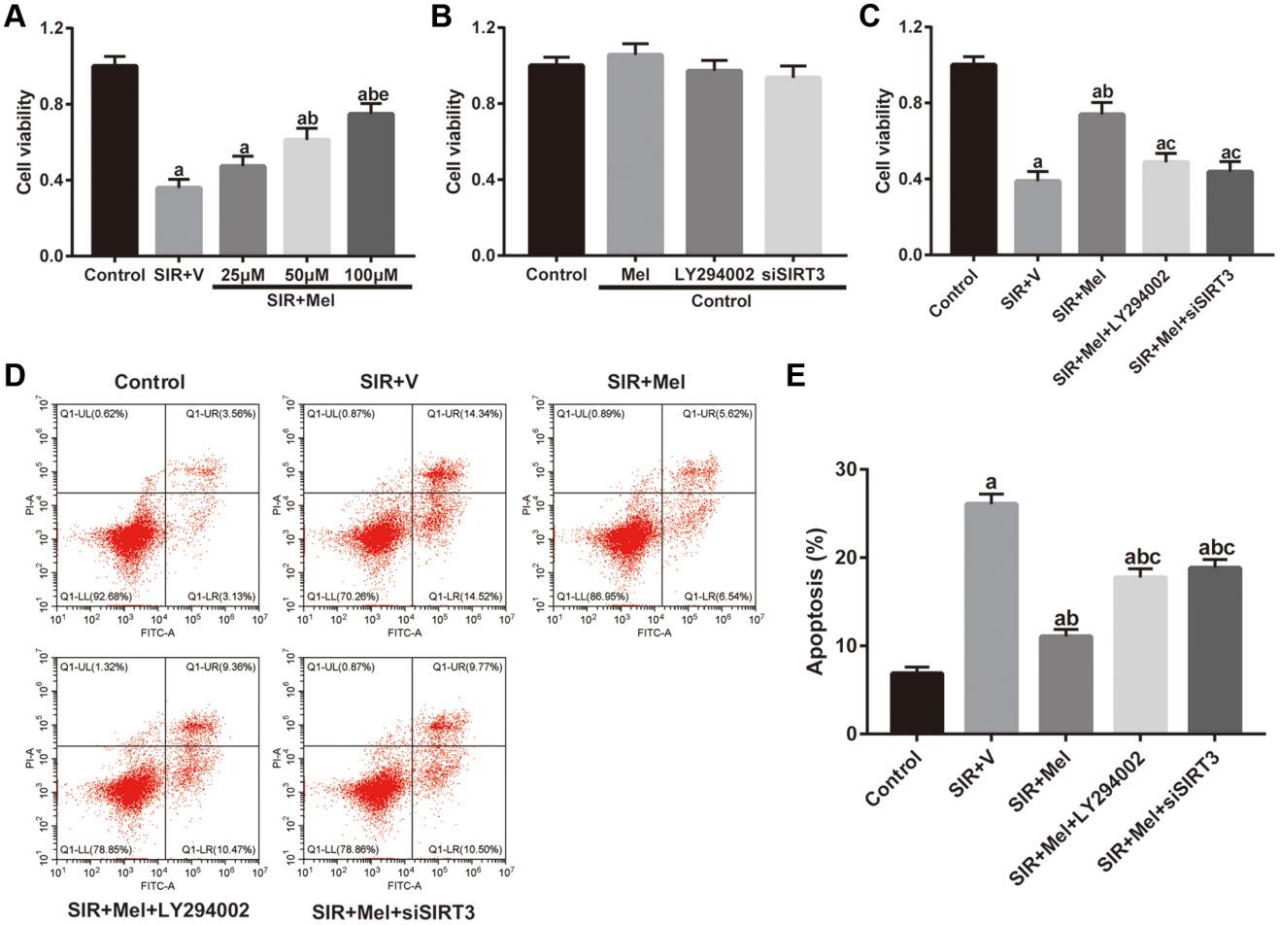
**Effects of melatonin on cell viability and apoptosis in high glucose-treated HT22 cells after SIR operation.** (**A**–**C**) Cell viability was measured by the CCK8 kit. (**D**) Representative images for apoptosis assessed by flow cytometry. (**E**) Quantitative analysis of the levels of apoptosis. Data were presented as the mean ± SEM (*n* = 6). ^a^*p* < 0.05 vs. control group; ^b^*p* < 0.05 vs. SIR+V group; ^c^*p* < 0.05 vs. SIR+Mel group; ^e^*p* < 0.05 vs. Mel (25 μM).

We further performed *in vitro* studies using murine hippocampal neuron cell line HT22 to investigate whether melatonin directly protects against CIR injury and to illuminate the underlying mechanism. As shown in Figure 5A, SIR-injured HT22 cells were treated with different concentrations of melatonin (25 μM, 50 μM, and 100 μM) and cell viability was then tested. Melatonin notably increased the cell viability of SIR-injured HT22 cells in a dose-dependent manner. The cytoprotective effect of melatonin was most obvious at 100 μM and 100 μM was then selected for further mechanistic investigations. Furthermore, the effects of melatonin, LY294002 and siSIRT3 treatments were evaluated in control cells. As shown in Figure 5B, melatonin, LY294002, and siSIRT3 treatments had no significant effects on the cell viability of high glucose-treated control HT22 cells, respectively. However, LY294002 or siSIRT3 treatment significantly reduced melatonin-mediated increase of cell viability in high glucose-treated HT22 cells following SIR injury (Figure 5C). Additionally, the apoptosis level was markedly reduced by melatonin pretreatment compared with that in the SIR+V group. Nevertheless, the anti-apoptosis effects mediated by melatonin observed in the SIR + Mel group were markedly attenuated by either LY294002 or siSIRT3 (Figure 5D and 5E). These results provided evidence that Akt signaling and SIRT3/SOD2 signaling are key mediators of the cytoprotective actions of melatonin.

### Effects of Akt signaling and SIRT3/SOD2 signaling on melatonin-mediated suppression of mitochondrial oxidative stress against SIR injury in high glucose-treated HT22 cells

**Figure 6:**
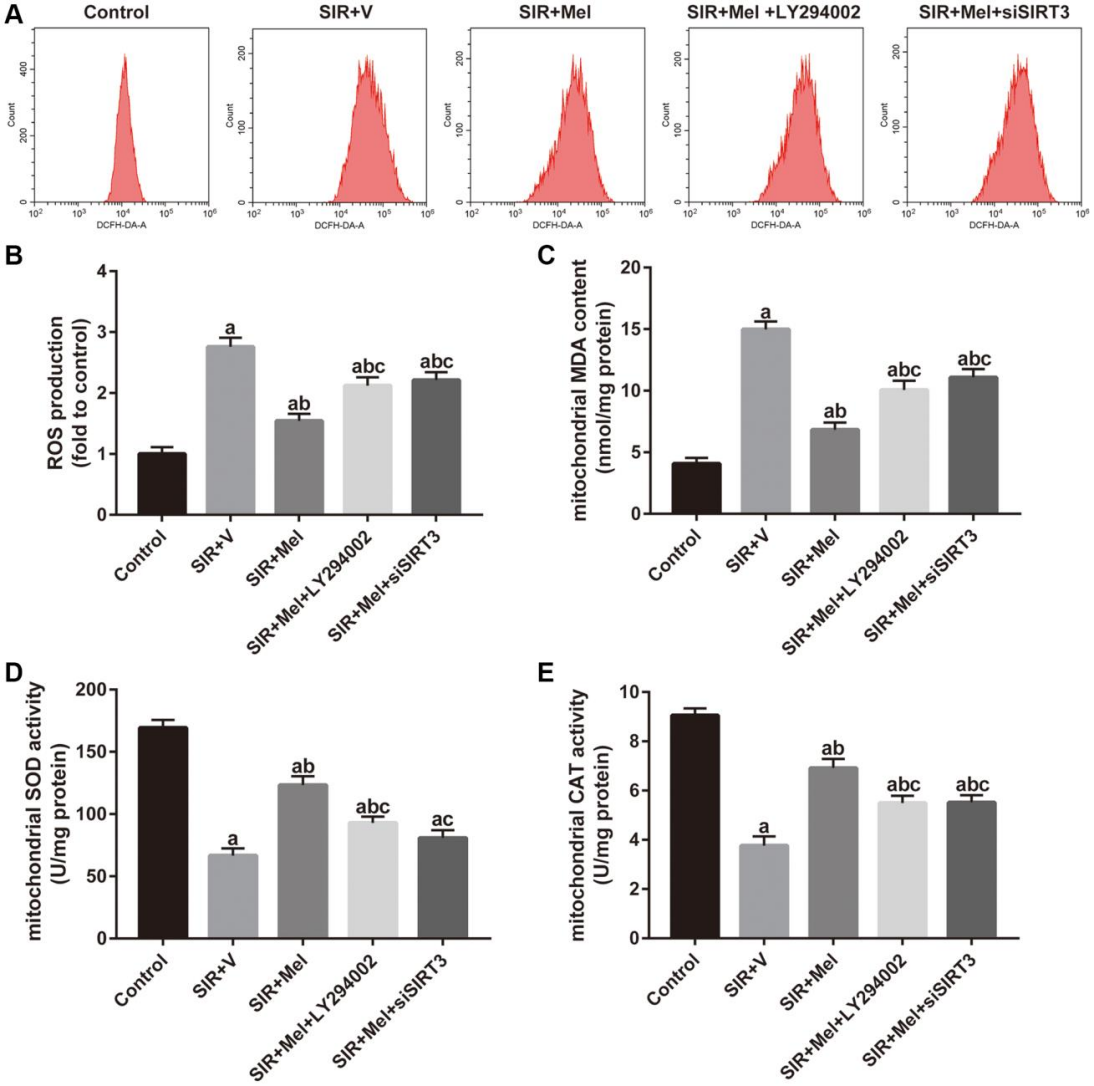
**Effects of LY294002 and siSIRT3 on the melatonin-mediated reduction in mitochondrial oxidative stress in high glucose-treated HT22 cells with SIR injury.** (**A**) Representative images for ROS generation assessed by flow cytometry. (**B**) Quantitative analysis of ROS production. (**C**–**E**) Quantitative analysis of levels of mitochondrial MDA, SOD and CAT were quantified by using commercial kits. Data were presented as the mean ± SEM (*n* = 6). ^a^*p* < 0.05 vs. control group; ^b^*p* < 0.05 vs. SIR+V group; ^c^*p* < 0.05 vs. SIR+Mel group.

As shown in Figure 6A and 6B, melatonin markedly decreased intracellular ROS production and the alleviative effect was largely weakened by either LY294002 or siSIRT3. Simultaneously, mitochondrial MDA generation significantly decreased in the melatonin-treated group, accompanied by increased activity of SOD and CAT. However, the mitigatory effects were also blunted by LY294002 or siSIRT3 (Figure 6C–6E). These data showed that melatonin could attenuate mitochondrial oxidative damage against SIR injury via Akt and SIRT3/SOD2 signaling in high glucose-treated HT22 cells.

### Effects of Akt signaling and SIRT3/SOD2 signaling on melatonin-mediated improvements in mitochondrial impairments in HT22 cells following SIR injury

**Figure 7:**
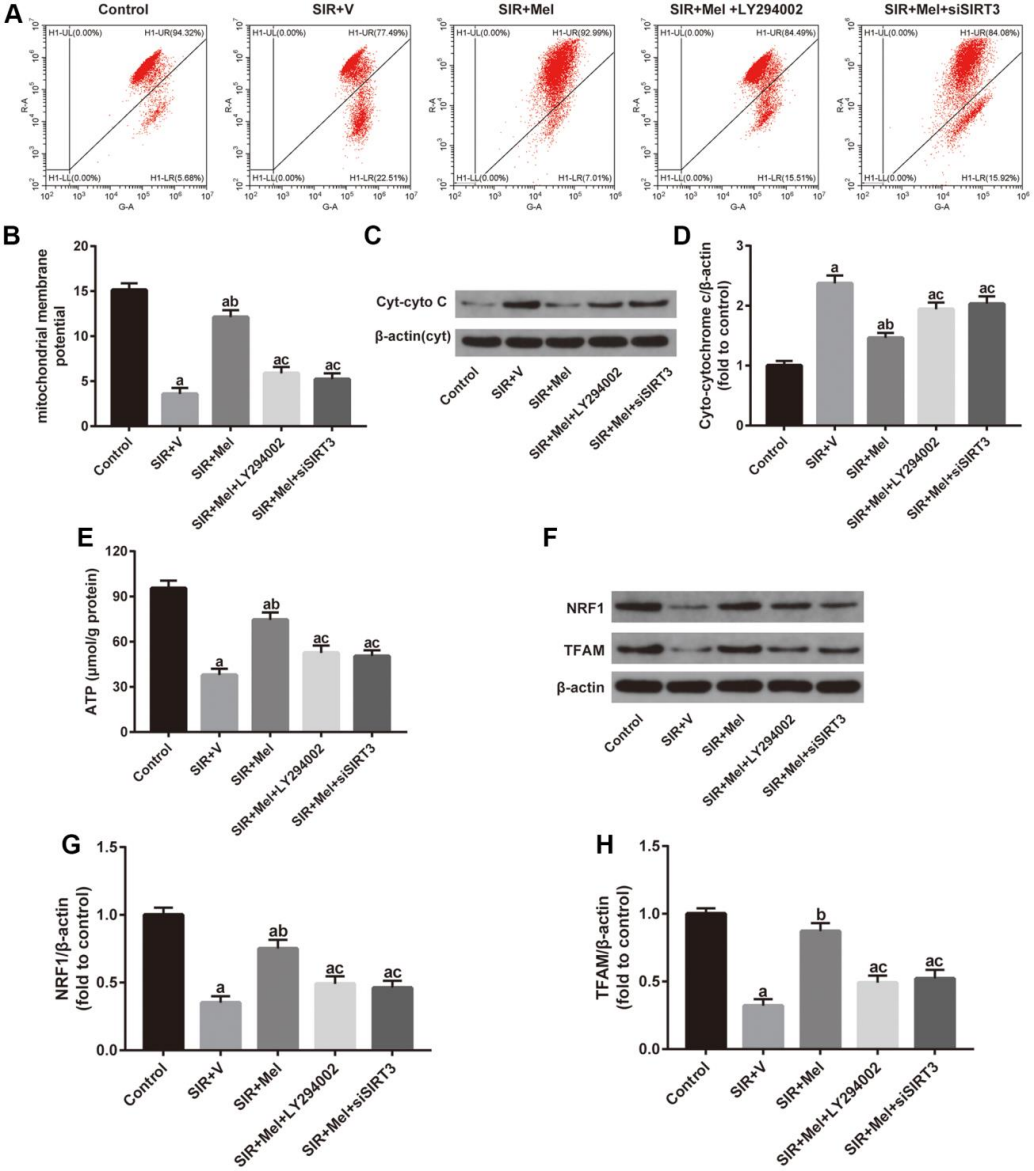
**Effects of LY294002 or siSIRT3 on the melatonin-mediated improvement in mitochondrial defects in high glucose-treated HT22 cells with SIR injury.** (**A**) Representative images for mitochondrial membrane potential (MMP) assessed by flow cytometry. (**B**) Quantitative analysis of MMP. (**C**) Representative images for Cyt-cyto C expression detected by Western blot. (**D**) Quantitative analysis of the Cyt-cyto C protein levels. (**E**) Quantitative analysis of mitochondrial ATP content. (**F**) Representative images for NRF1 and TFAM expression detected by Western blot. (**G**–**H**) Quantitative analysis of the NRF1 and TFAM levels. Data were presented as the mean ± SEM (*n* = 6). ^a^*p* < 0.05 vs. control group; ^b^*p* < 0.05 vs. SIR+V group; ^c^*p* < 0.05 vs. SIR+Mel group.

To further confirm the beneficial role of melatonin, we measured mitochondrial membrane potential (MMP), an important parameter of the mitochondrial function used as an indicator of ATP synthesis [39], and mitochondrial ATP content in our *in vitro* study. As shown in Figure 7A, 7B and 7E, melatonin treatment significantly ameliorated SIR induced-reduction of MMP and ATP levels and these effects were largely reversed by either LY294002 or siSIRT3. Additionally, western blot analysis showed that expression of Cyt-cyto C was noticeably reduced while the expression of NRF1 and TFAM was increased in the melatonin-treated group. However, LY294002 or siSIRT3 also blunted these effects (Figure 7C, 7D and 7F–7H). These data suggested that melatonin could alleviate the impairments in mitochondrial function and biogenesis in high glucose-treated HT22 cells. Importantly, Akt and SIRT3/SOD2 signaling mediated this action.

### Relationship between the Akt signaling and the SIRT3/SOD2 pathway in the neuroprotective effects of melatonin

**Figure 8:**
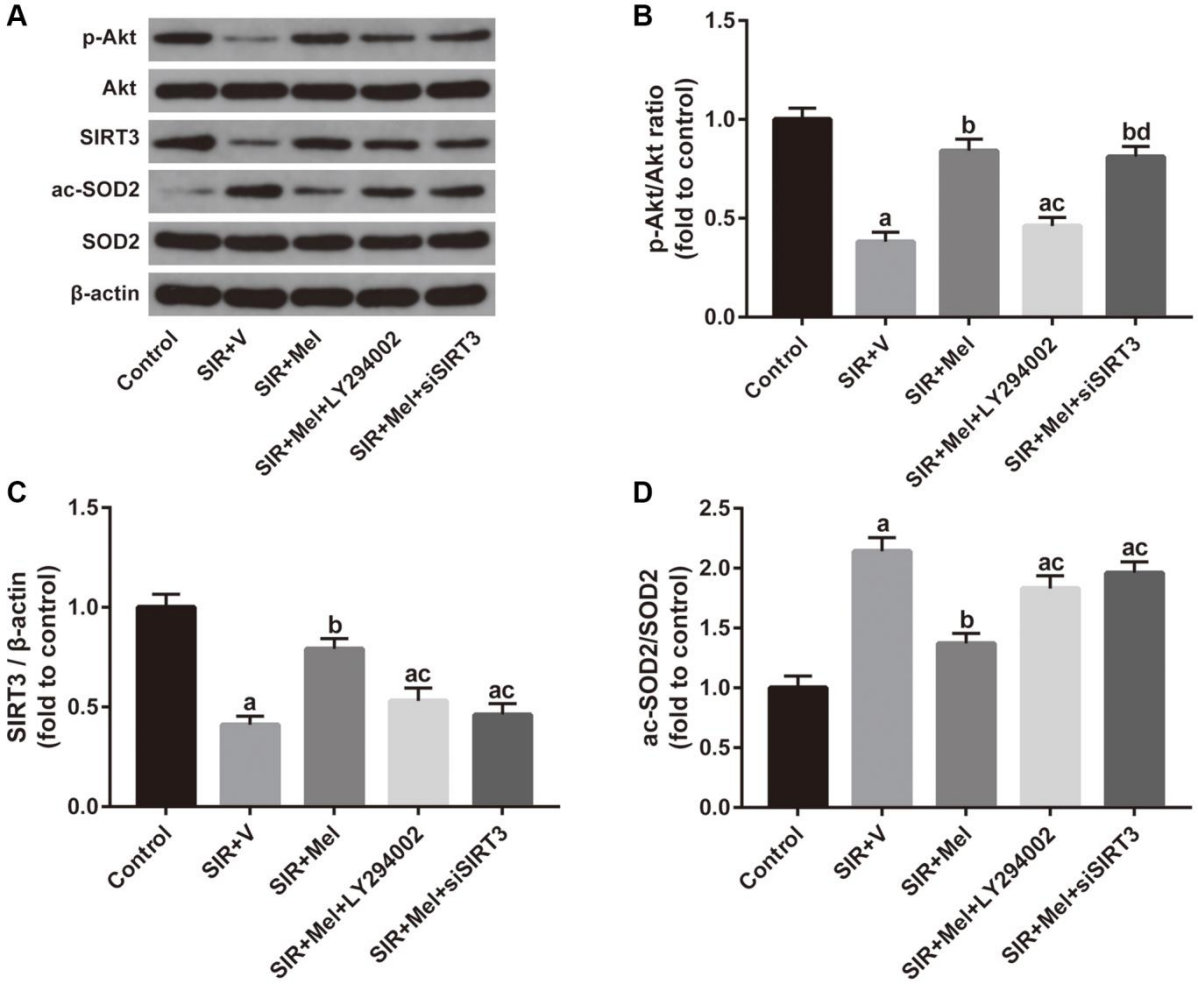
**Correlation between the Akt signaling and the SIRT3/SOD2 signaling in mediating the neuroprotective actions of melatonin.** (**A**) Representative images for Akt phosphorylation, SIRT3 expression, and SOD2 acetylation detected by Western blot. (**B**–**D**) Quantitative analysis of the ratio of p-Akt/Akt, expression of SIRT3, and the ratio of ac-SOD2/SOD2. Data were presented as the mean ± SEM (*n* = 6). ^a^*p* < 0.05 vs. control group; ^b^*p* < 0.05 vs. SIR+V group; ^c^*p* < 0.05 vs. SIR+Mel group; ^d^*p* < 0.05 vs. SIR+Mel+LY294002 group.

Finally, we focused on the correlation between the Akt signaling and the SIRT3/SOD2 signaling. Both *in vivo* and *in vitro* experiments revealed that inhibiting Akt signaling with LY294002 noticeably weakened melatonin-induced increase of p-Akt/Akt ratio and SIRT3 expression and obviously impaired melatonin-induced decrease of SOD2 acetylation. Additionally, our *in vitro* study further found that siSIRT3 markedly attenuated melatonin-mediated upregulation of SIRT3 expression and apparently weakened melatonin-induced reduction of the ac-SOD2/SOD2 ratio but failed to change the ratio of p-Akt/Akt. (Figure 8). Collectively, these data suggested that Akt might function as an upstream regulator of SIRT3/SOD2 signaling in mediating the neuroprotective effects of melatonin against CIR injury in diabetic states.

## DISCUSSION

Our present results provide evidence that melatonin protects the brain against CIR injury in the diabetic state by alleviating mitochondrial impairments *in vivo* and *in vitro*. We found that (a) melatonin treatment reduced cerebral infarct volume, neurological deficits, and brain edema after CIR injury in diabetic mice, as well as increase the cell viability in high glucose-treated HT22 cells following SIR injury; (b) melatonin attenuates CIR-induced mitochondrial oxidative damage and dysfunction in the diabetic state; (c) the neuroprotective effects of melatonin were mediated, at least in part, by activating the Akt-SIRT3-SOD2 signaling pathway. These results provide a novel insight into the mechanism of diabetes complicated with CIR injury and offer a potentially beneficial approach to ameliorate brain damage induced by CIR in the diabetic population.

Ischemic stroke is the second leading cause of death worldwide and the major cause of chronic disability in adults, causing approximately 6.2 million deaths each year [40, 41]. Diabetes, with its soaring prevalence worldwide, has been demonstrated to be an independent risk factor for stroke, increasing the risk of stroke by 1.8 to 6 times [42]. In diabetic patients, CIR injury is associated with elevated vulnerability to disability and death and the long-term prognosis is also much worse than non-diabetic individuals [43, 44]. Disappointingly, the preventive or therapeutic approaches against diabetic CIR injury, such as rigorous glycemic control and thrombolysis, carry major risk accompanying theoretical but unrealized protective effects [45] Therefore, it is highly necessary to explore safe and effective therapies to reduce the incidence of cerebrovascular events and alleviate CIR injury in diabetic patients.

Mitochondria, very susceptible to any insult, can trigger a series of catabolic reactions [46]. In recent years, mitochondria have received increasing attention as therapeutic targets for neurodegenerative diseases due to their key roles in the production of ATP and ROS, which are key mediators of cellular signal transduction and energy homeostasis [47, 48]. Compared to other cell types, neurons have higher energy expenditure and little energy reserve [49]. During CIR, the substrates needed for energy production are rapidly depleted, and mitochondria are severely impaired [47, 50]. Prolonged hyperglycemia in diabetes deteriorated energy metabolism and ROS homeostasis, further caused damage in mitochondria, which can lead to the failure of cellular pumps and cause cytotoxic edema and cell death [19, 51]. Additionally, the ROS-scavenging system and mitochondrial biogenesis have been reported to play a crucial role as an endogenous protective mechanism during CIR [52]. In line with these studies, our present study demonstrated that CIR injury increased mitochondrial oxidative damage, impaired structure and functions of mitochondria and disturbed mitochondrial biogenesis in diabetic mice and high glucose-treated HT22 cells, suggesting that mitochondrial impairments may be the key mechanism of diabetic CIR injury.

Melatonin, a pleiotropic hormone synthesized by the pineal gland at night, regulates a variety of physiological functions in numerous organs [53]. Owing to its highly lipophilic properties that can easily cross most biological cell membranes along with its low toxicity to humans, melatonin has attracted great clinical interest [54, 55]. Previously, we have demonstrated that melatonin attenuated acute kidney ischemia-reperfusion injury in diabetic rats [56]. Additionally, numerous studies have documented a pronounced protective effect of melatonin against CIR injury in non-diabetic animals [47, 57, 58]. However, the neuroprotective mechanisms exerted by melatonin in diabetes complicated with CIR injury remain unclear. Interestingly, mitochondria are the main sites for melatonin synthesis in various cells, including neurons [59, 60]. Zhou et al. demonstrated that melatonin promoted osteogenesis by ameliorating mitochondrial oxidative stress [34]. Notably, Yang et al. reported that in a non-diabetic CIR mice model, melatonin treatment attenuates CIR injury by reducing CIR-induced mitochondrial dysfunction [47]. These studies suggest that mitochondria may be a key target of melatonin in various diseases. Consistent with these findings, we found that under the diabetic state, melatonin not only alleviated the mitochondrial oxidative damage but also significantly ameliorated the impairments in the mitochondrial structure and function. Notably, mitochondrial biogenesis has been shown to help mitigate oxidative stress-induced detrimental consequences and has been recognized as a new component of the central nervous system repair mechanism [61, 62]. As expected, we also observed that the melatonin-treated group showed noticeably upregulated expressions of the two mitochondrial biogenesis factors, NRF1 and TFAM. Increased activity of endogenous antioxidant enzymes SOD and CAT in our study may be the result of increased mitochondrial biogenesis [63].

Another novel finding of this study is that we proved the roles of Akt-SIRT3-SOD2 signaling in melatonin’s neuroprotective effects in diabetes complicated with CIR injury. SOD2, a major mitochondrial oxidative scavenging enzyme, plays essential roles in the regulation of ROS balance [64]. The activity of SOD2 is tightly regulated by acetylation at its lysine residues and is inversely proportional to its acetylation [65, 66]. SIRT3, the most robust mitochondrial deacetylase, has been reported to function as a key regulator of SOD2 activity by direct deacetylation of the SOD2 gene [66]. Recently, Liu et al. found that SIRT3 repression results in SOD2 acetylation, leading to SOD2 inactivation, which enhanced high glucose-induced oxidative stress and cytotoxicity in endothelial cells [67]. Katwal et al. demonstrated a protective effect of SIRT3 against hepatic ischemia-reperfusion injury via regulation of its downstream mediator SOD2 [68]. Li and colleagues showed that phosphocreatine attenuated liver injury by the SIRT3/SOD2 pathway mediated mitochondrial protection [35]. In line with these studies, the downregulation of SIRT3 expression, the decrease in SOD2 deacetylation. and the increase of mitochondrial impairments occurred in both diabetic brain and high glucose treated-HT22 cells following CIR operation in our present study. Besides, melatonin treatment significantly ameliorated the SIRT3 expression and SOD2 deacetylation and alleviated the mitochondrial impairments. However, these effects were largely weakened by the SIRT3 specific inhibitor 3-TYP or SIRT3 siRNA. Moreover, inhibition of SIRT3/SOD2 signaling noticeably blunted the neuroprotective action of melatonin as well, indicating that the SIRT3-SOD2 signaling mediated mitochondrial protection is vital to the neuroprotective actions of melatonin on CIR-injured brain or SIR-treated HT22 cells in the hyperglycemic state.

Indeed, Akt and SIRT3-SOD2 signaling are both essential for preserving mitochondrial function [35, 69]. Recently, Song et al. reported that melatonin upregulated SIRT3 expression through the Akt signaling pathway in TNF-α-treated hepatocytes [36]. It has also been found that the SIRT3 expression was regulated by the phosphatidylinositol 3-kinase/Akt signaling in Rg3(S) treated human diploid fibroblasts and sodium fluoride-treated hepatocytes [70, 71]. Collectively, the data suggested that Akt may function as the upstream regulator of SIRT3. Consistently, our present study demonstrated that melatonin alleviated mitochondrial impairments and triggered Akt phosphorylation, SIRT3 upregulation, and SOD2 deacetylation while inhibition of Akt significantly weakened the beneficial effects of melatonin and downregulated Akt phosphorylation, SIRT3 expression, and SOD2 deacetylation. Moreover, it is noteworthy that inhibition of SIRT3 largely attenuated the protective effects of melatonin and blunted the elevation of SOD2 deacetylation mediated by melatonin treatment without affecting the Akt phosphorylation. Therefore, we conclude that melatonin reduces mitochondrial defects by activating the Akt-SIRT3-SOD2 axis, thereby reducing CIR damage in diabetic states.

Recent studies demonstrated that the neuroprotective effects of melatonin in CIR injury animal models were mediated through receptor-dependent or receptor-independent manners [59, 60, 72]. It will contribute to further revealing the underlying mechanisms if we could identify whether the protective effects of melatonin are mediated by its receptors or it is a receptor-independent activity in diabetic mice with CIR injury by using receptor agonists and antagonists in our future study. Additionally, experiments using Akt-and SIRT3-deficient animals would also be helpful to further confirm the underlying mechanisms.

Taken together, this study provides the first evidence for the potential neuroprotective effects of melatonin in diabetes complicated with acute ischemic stroke. We found that melatonin-mediated amelioration of CIR injury in diabetic states can be attributed to its mitochondrial protective actions. More importantly, we clarified the critical role of the Akt-SIRT3-SOD2 signaling pathway in melatonin’s neuroprotective actions. These results suggested that melatonin treatment might be a promising therapeutic strategy for diabetic patients with ischemic stroke.

## MATERIALS AND METHODS

### Animals

Specific-pathogen-free (SPF) male C57BL/6J mice (4–5 w, 18–20g) were purchased from Beijing Vital River Laboratory Animal Technology Co., Ltd. (Beijing, China). All mice were housed in the Animal Center of Renmin Hospital of Wuhan University under pathogen-free conditions with a 12-hour light/12-hour dark cycle (lights on at 07:00) at 22–24°C and fed a regular pellet diet ad libitum. All experimental protocols were approved by the Laboratory Animal Welfare & Ethics Committee (IACUC) of Wuhan University (issue no. WDRM20151210), and experimental processes were performed according to the National Institutes of Health Guide for the Care and Use of Laboratory Animals. All operations and detection were performed during the morning hours to prevent the influence of the time-of-day variation on mice.

### Reagents

Melatonin, streptozotocin (STZ), and 2,3,5-triphenyl tetrazolium chloride (TTC) were obtained from Sigma-Aldrich (St. Louis, MO, USA). LY294002 and 3-TYP were purchased from MedChemExpress (MCE; Monmouth Junction, NJ, USA). Dulbecco’s Modified Eagle Medium (DMEM) and penicillin/streptomycin were obtained from GENOM (Hangzhou, China), fetal bovine serum (FBS) were bought from TIANHANG (Zhejiang, China). The mitochondrial membrane potential assay kit with JC-1 and the ROS Assay Kit were got from Beyotime (Shanghai, China). Cell Counting Kit-8 (CCK-8) was obtained from Absin (Shanghai, China). Kits for detecting malondialdehyde (MDA) content, superoxide dismutase (SOD) activity, catalase (CAT) activity, and ATP content and Mitochondria Isolation Kit were purchased from the Nanjing Jiancheng (Nanjing, Jiangsu, China). The Annexin V-FITC Apoptosis Detection Kit was obtained from KeyGEN (Nanjing, Jiangsu, China). Primary antibodies against p-Akt, Akt, cytochrome c (cyto-C), and β-actin, as well as the secondary antibodies, were all purchased from Cell Signaling Technology (CST; Boston, MA, USA). Primary antibodies against SIRT3, SOD2 (acetyl K68) (ac-SOD2), SOD2, TFAM, TFAM were purchased from Abcam (Cambridge, MA, USA).

### Diabetic model establishment

After one week’s acclimation, the mice diabetic model was constructed as previously described [73]. Mice were fasted overnight and received an intraperitoneal injection (i.p.) of STZ at a dose of 50 mg/kg for five consecutive days. One week later, blood glucose concentrations in samples obtained from the tail vein were measured by a glucometer (Johnson & Johnson, USA). Mice with random blood glucose concentration > 16.7 mmol/L (300 mg/dL) were considered diabetes.

### Experimental design

All diabetic mice were randomly divided into the following groups (*n* = 15): (a) Sham group: mice underwent the sham operation and were treated with vehicle; (b) Vehicle group: mice underwent the CIR operation and were treated with vehicle; (c) Mel group: mice underwent the CIR operation and were treated with melatonin (10 mg/kg i.p., immediately after induction of ischemia and at reperfusion onset); (d) Mel+3-TYP group: mice were subjected to the CIR operation, and pretreated with melatonin and 3-TYP (50 mg/kg i.p., every 2 days for a total of three times); (e) Mel +LY294002 group: mice were subjected to the CIR operation and administered with melatonin and LY294002 (15 nmol/kg, injected by tail vein 30min before the ischemia). The dosage regimen of melatonin, 3-TYP, and LY294002 were based on previous studies [47, 57, 58, 74].

### Cerebral ischemia-reperfusion (CIR) injury model construction

The CIR injury model (or the sham operation) was constructed in mice four weeks after established diabetes. CIR injury was induced by middle cerebral artery occlusion (MCAO) in the mice using a suture embolism, as previously described [75]. Briefly, a face mask was applied to the mice and was connected to a gaseous anesthetic system. The depth of anesthesia was monitored by checking toe pinch responses. All the mice were deeply anesthetized with 5% isoflurane, followed by 60 min occlusion of the left middle cerebral artery (MCA) with a 6–0 suture. The suture was then carefully removed to initiate reperfusion. Sham-operated mice underwent the same surgical procedures without placing the suture in the MCA. During surgery, the mice's body temperature was maintained at 37°C with a homeothermic heating pad. To minimize the risk of pain, EMLA cream (lidocaine 2.5% and prilocaine 2.5%) was externally applied for analgesia using sterile swabs to cover the incision site soon after the surgery.

### The neurological deficit, cerebral infarct volume, and brain water content assessment

24 h after reperfusion, the neurological deficit scores were evaluated as described previously [23]. Each mouse was scored by three examiners who were blinded to the treatment protocol. The score ranged from 0 (no motor deficits) to 4 (critical). After being evaluated for the neurological deficit, the cerebral infarct volume was measured by TTC staining and analyzed by ImageJ v1.61 (National Institutes of Health, Bethesda, MD, USA) according to the indirect method and corrected for edema by comparing the volume of the ischemic and nonischemic hemispheres as previously described [23]. The infarct volume was presented as a percentage of the whole volume. After obtaining the photos of the infarct area, brain edema was assessed by the wet/dry method [47]. Briefly, the wet weight of the brain slices was quantified and then was dried at 105°C for 48 h to determine the water content. The brain water content was calculated by using the following formula: (wet weight-dry weight)/wet weight × 100%.

### Transmission electron microscopy (TEM) observation

Fragments of 1 mm^3^ of periinfarct tissue in the cerebral cortex isolated from mice brain were collected 24 h after reperfusion and immobilized overnight in 2.5% glutaraldehyde at 4°C. The tissue samples were washed, fixed, dehydrated, embedded, and cured with buffer solution, and then cut into ultra-thin sections using an ultra-thin slicer. The ultrastructure of the mitochondria was scanned using a TEM (Hitachi, Japan) at 12.0k magnification.

### Simulated ischemia-reperfusion (SIR) and cell treatment

Murine hippocampal neuron cell line HT22 (Procell, Wuhan, China) were cultured in DMEM (25 mM glucose) supplemented with 10% FBS and 1% penicillin/streptomycin and maintained at 37°C in a humidified incubator containing 5% CO2. HT22 cells were cultured in a high-glucose medium (50 mM glucose) for 8 h before SIR treatment and during the entire reperfusion period to mimic the *in vivo* diabetic model. SIR injury was initiated by incubating HT22 cells for 6 h in a hypoxic incubator (Binder, CB-210 hypoxia workstation) with 1% O_2_, 5% CO_2_, and 94% N_2_. Subsequently, cultures were returned to the normoxic incubator for 24 h, corresponding to the reperfusion period [76, 77]. To select the appropriate concentration of melatonin, HT22 cells were pretreated with melatonin at a range of concentrations (25, 50, 100 μM) for 4 hours in SIR-injured HT22 cells. Cell viability assays suggested that the pro-survival effect of melatonin was most noticeable with a concentration of 100μM. Consequently, a dose of 100 μM was chosen for the subsequent experiments. Then, high glucose-incubated HT22 cells were randomly divided into five groups as follows: the Control group, the cells were pretreated with the vehicle without SIR treatment; the SIR+V group, the cells were pretreated with the vehicle for 4 hours and then exposed to SIR treatment as mentioned above; the SIR+Mel group, the cells were pretreated with melatonin (100 μM) for 4 hours and then exposed to SIR treatment; the SIR+Mel+LY294002 group, the cells were treated with melatonin (100 μM) and LY294002 (10 μM) for 4 hours before SIR treatment; the SIR+Mel+siSIRT3 group, the cells in which were transfected with the SIRT3-specific siRNA, then treated with melatonin (100 μM) for 4 hours before SIR treatment. The doses of LY294002 and siSIRT3 were chosen based on previous studies and the manufacturers’ instructions [78–80].

### Small interfering RNA (siRNA) transfection

SIRT3 siRNA duplex solution, transfection reagent, and medium were all obtained from RiboBio (Guangzhou, China). HT22 cells were transfected with either 100 nM SIRT3-targeting small siRNA (siSIRT3) or a control nonspecific siRNA (si-control) following the manufacturer's instruction as described previously [63, 79]. After transfection for 72 h, the cells were subjected to various treatments or measurements as described above.

### Cell viability assay

Cell viability was determined by the CCK-8 according to the manufacturer’s protocol. Briefly, HT22 cells were seeded in 96-well plates and pretreated with various conditions as described above, followed by incubating with 10 μL CCK-8 solution for 4 hours, and the absorbance at 450 nm was measured using a microplate reader. The results were presented as the fold of control.

### ROS assessment

For the determination of ROS generation, brain sections were incubated with 10 μmol/L DHE in the dark for 30 min at 37°C. Sections were then washed in PBS for 3 × 10 min, dried off, and then mounted with DAPI and coverslip. The brain tissue slides were observed with a fluorescence microscope (Nikon Eclipse C1), and the intensity of DHE fluorescence in brain sections was analyzed by Image Pro-Plus 6.1 analysis system (Media Cybernetics Inc., Silver Spring, MD, USA). The results were presented as fold change from the sham control [81]. Intracellular ROS production following SIR in HT22 cells was measured by the ROS Assay Kit using flow cytometry (Beckman Coulter CytoFLEX) according to the manufacturers’ instructions. The results were presented as fold change from the control group [82].

### Mitochondria and cytosol fraction isolation

The isolation of mitochondrial/cytosol fraction was performed using the mitochondria isolation kit according to the manufacturer's protocol. Briefly, the brain tissues or cells were washed and homogenized using lysis buffer, and then were centrifuged at 800 g for 5 min at 4°C. Solution A was added to the collected supernatant and centrifuged at 15,000 g for 10 min at 4°C. The obtained supernatant was the cytosolic fraction, which was transferred to another tube. The resulting sediment, which consisted of mitochondrial fraction, was re-suspended in rinsing solution and further centrifuged at 15,000 g for 10 min. The supernatant was removed and the mitochondrial precipitation was then resuspended with a storage solution or an appropriate buffer.

### Mitochondrial oxidative stress and functional evaluation

The levels of oxidative stress markers (MDA content, SOD activity, CAT activity) and ATP production in the mitochondria were assessed by correspondingly commercially available kits as described previously [37, 74, 75]. The MMP was assayed by flow cytometry using the mitochondrial membrane potential assay kit with JC-1. In brief, HT22 cells were incubated with a JC-1 solution for 20 min at 37°C in the dark and were then collected for subsequent flow cytometry analysis. The results are expressed as a relative red/green fluorescence ratio [83]. Additionally, we also measured the Cyt-cyto C expression to evaluate the mitochondrial integrity and apoptosis [38].

### Flow cytometry and apoptosis analysis

Cell apoptosis was analyzed using an Annexin V-FITC Apoptosis Detection Kit according to the manufacturer's protocols. HT22 cells at 80% confluency were harvested using 0.25% trypsin for 5 min at 37°C and washed twice with PBS. Following centrifugation at 2000 rpm for 5 min at 4°C, cells were resuspended in a solution containing Annexin V-FITC and propidium iodide for 15 min at room temperature. Subsequently, the cells were analyzed with a flow cytometer (Beckman Coulter CytoFLEX).

### Western blot analysis

Western blot analysis was performed as described previously [75]. Briefly, proteins of brain tissue and HT22 neurons were prepared and separated on SDS-PAGE gels. Then, they were transferred to PVDF membrane and incubated overnight at 4°C with p-Akt, Akt, SIRT3, SOD2 (acetyl K68) (ac-SOD2), SOD2, NRF1, TFAM, cytochrome c, and β -actin antibodies (1:1000 dilution). Then, the membranes were washed and probed with the secondary antibodies for 1 hour at room temperature. The β-actin antibody was used as an internal control. The blot bands were quantified by ImageJ v1.61 (National Institutes of Health, Bethesda, MD, USA).

### Statistical analysis

All the results are shown as the means ± standard error of the mean (SEM). Data were analyzed by one-way analysis of variance (ANOVA) followed by Tukey’s post hoc test. *p* < 0.05 were considered to be statistically significant. The statistical analyses were performed using SPSS 18.0 (SPSS Inc., Chicago, IL, USA).
